# Commentary: The Synergistic Role of Irreversible Electroporation and Chemotherapy for Locally Advanced Pancreatic Cancer

**DOI:** 10.3389/fonc.2022.955444

**Published:** 2022-09-15

**Authors:** Vincenza Granata, Raffaele Palaia, Francesco Izzo

**Affiliations:** ^1^ Division of Radiology, “Istituto Nazionale Tumori IRCCS Fondazione Pascale – IRCCS di Napoli”, Naples, Italy; ^2^ Division of Hepatobiliary Surgical Oncology, “Istituto Nazionale Tumori IRCCS Fondazione Pascale – IRCCS di Napoli”, Naples, Italy

**Keywords:** electrochemotherapy (ECT), irreversible electroporation (IRE), pancreas, bleomycin, chemotherapy – oncology

**Letters to the Editor**

We read with interest the article from Dr. Gyftopoulos and colleagues in *Frontiers in Oncology* (1) in which they assessed irreversible electroporation (IRE) as an interesting tool in the treatment of locally advanced pancreatic cancer (LAPC). We congratulate the authors on their accuracy in data presentation about IRE in LAPC (2–4). However, we would like to clarify the different concepts of IRE and electrochemotherapy (ECT) and to report recent results on ECT in LAPC. Reversible and irreversible electroporation are two different ways of applying the electric field with the aim of permeabilizing the cell membrane and obtaining different effects: in the first case, the transient permeabilization of the cell membrane allows one to facilitate the administration of the drug, while in the second case, the irreversibly permeabilized cell membrane will subsequently undergo cell death. Therefore, the correct use of the term “electrochemotherapy” (ECT) is associated with a combined therapy based on electric pulses and drug, known as combining chemotherapeutic drugs with an electrical field that determines a transient increase of cell permeability (reversible electroporation), allowing uptake of chemotherapy into tumor, using low doses of drugs, and reducing chemotherapy cytotoxic effects (4, 5). Therefore, differently from what was reported by Dr. Gyftopoulos and colleagues (1), IRE cannot be defined using the term “ECT”, and although both IRE and ECT can be safely used to treat LAPC patients, these techniques are profoundly different (6–10). The drugs used in ECT is defined by *ex vivo* and *in vivo* experiments and clinical trials where these drugs (including bleomycin and cisplatin) can accumulate in the cancer cells treated with reversible electroporation (RE) and, hence, achieve a local tumor ablation effect when a lower-than-conventional chemotherapeutic dose of drug is administered systemically or locally. ECT is considered a local tumor treatment, in which a drug is usually given once or a few times concomitant with RE if necessary, whereas chemotherapy is systemic therapy, which is often given repetitively through several drugs combined with multiple cycles. Therefore, the synergistic mechanism of IRE and chemotherapy, which should not be seen/confused with that of ECT, is likely a combination of the cytoreductive effect by one-time IRE local tumor ablation and systemic cancer cell inhibition/clearance by chemotherapy. Moreover, there is no such evidence that other chemo drugs can be automatically considered for ECT, or when co-implementation of impulse chemotherapy (RE or IRE) occurs, drug release facilitated by electro-pulse in cancer cells (“ECT” effect) must happen. Therefore, drugs that claim to have a synergistic effect with EP (defined by EP-facilitated drug into cancer cells) must be validated by preclinical and clinical experiments first.

Some authors have evaluated the feasibility and effectiveness of electrochemotherapy on deep tumors (6–18). In our previous study, we showed that electrochemotherapy on pancreatic tumor can be performed safely and feasibility [6]. No side effects or major complications, no clinically relevant elevation of amylase and lipase levels, and no evidence of clinical pancreatitis were observed in the LAPC patients treated with ECT. Although ECT has been shown to be a promising technique for cancer treatment, the question how to assess the response of the treated tumor still exists. ECT potentiates the cytotoxic effect of chemotherapy, and therefore, the CHOI criteria would appear to be more suitable for early treatment evaluation (8). We demonstrated that a local disease control (partial response) was obtained according to the CHOI criteria in 18/18 (100.0%) patients treated with ECT (8).

In conclusion, we believe that the readers of *Frontiers in Oncology* should know that the term “IRE” should not be confused with the term “electrochemotherapy”, which is reserved to a combination of low doses of chemotherapeutic drugs with an electrical field that determines a transient increase of cell permeability (reversible electroporation) (13–18).

## Author contributions

All authors listed have made a substantial, direct, and intellectual contribution to the work, and approved it for publication.

## Conflict of interest

The authors declare that the research was conducted in the absence of any commercial or financial relationships that could be construed as a potential conflict of interest.

## Publisher’s Note

All claims expressed in this article are solely those of the authors and do not necessarily represent those of their affiliated organizations, or those of the publisher, the editors and the reviewers. Any product that may be evaluated in this article, or claim that may be made by its manufacturer, is not guaranteed or endorsed by the publisher.
